# Deeper insight into ferroptosis: association with Alzheimer’s, Parkinson’s disease, and brain tumors and their possible treatment by nanomaterials induced ferroptosis

**DOI:** 10.1080/13510002.2023.2269331

**Published:** 2023-11-27

**Authors:** Virendra Kumar Yadav, Nisha Choudhary, Amel Gacem, Rakesh Kumar Verma, Mohd Abul Hasan, Mohammad Tarique Imam, Ziyad Saeed Almalki, Krishna Kumar Yadav, Hyun-Kyung Park, Tathagata Ghosh, Pankaj Kumar, Ashish Patel, Haresh Kalasariya, Byong-Hun Jeon, Hassan Ali AlMubarak

**Affiliations:** a Department of Life Sciences, Hemchandracharya North Gujarat University, Patan, India; b Department of Physics, Faculty of Sciences, University 20 Août 1955, Skikda, Algeria; c Department of Biosciences, School of Liberal Arts & Sciences, Mody University of Science and Technology, Sikar, India; d Civil Engineering Department, College of Engineering, King Khalid University, Abha, Kingdom of Saudi Arabia (KSA); e Department of Clinical Pharmacy, College of Pharmacy, Prince Sattam Bin Abdulaziz University, Al Kharj, Saudi Arabia; f Faculty of Science and Technology, Madhyanchal Professional University, Bhopal, India; g Environmental and Atmospheric Sciences Research Group, Scientific Research Center, Al-Ayen University, Nasiriyah, Iraq; h Department of Pediatrics, Hanyang University College of Medicine, Seoul, Republic of Korea; i Department of Arts, School of Liberal Arts & Sciences, Mody University of Science and Technology, Sikar, India; j Department of Environmental Science, Parul Institute of Applied Sciences, Parul University, Vadodara, India; k Centre for Natural Products Discovery, School of Pharmacy and Biomolecular Sciences, Liverpool John Moores University, Liverpool, UK; l Department of Earth Resources and Environmental Engineering, Hanyang University, Seoul, Republic of Korea; m Division of Radiology, Department of Medicine, College of Medicine and Surgery, King Khalid University (KKU), Abha, Kingdom of Saudi Arabia

**Keywords:** Ferroptosis, neurodegenerative, Parkinson’s disorder, glioblastoma, glutathione

## Abstract

Ferroptosis is an emerging and novel type of iron-dependent programmed cell death which is mainly caused by the excessive deposition of free intracellular iron in the brain cells. This deposited free iron exerts a ferroptosis pathway, resulting in lipid peroxidation (LiPr). There are mainly three ferroptosis pathways viz. iron metabolism-mediated cysteine/glutamate, and LiPr-mediated. Iron is required by the brain as a redox metal for several physiological activities. Due to the iron homeostasis balance disruption, the brain gets adversely affected which further causes neurodegenerative diseases (NDDs) like Parkinson's and Alzheimer's disease, strokes, and brain tumors like glioblastoma (GBS), and glioma. Nanotechnology has played an important role in the prevention and treatment of these NDDs. A synergistic effect of nanomaterials and ferroptosis could prove to be an effective and efficient approach in the field of nanomedicine. In the current review, the authors have highlighted all the latest research in the field of ferroptosis, specifically emphasizing on the role of major molecular key players and various mechanisms involved in the ferroptosis pathway. Moreover, here the authors have also addressed the correlation of ferroptosis with the pathophysiology of NDDs and theragnostic effect of ferroptosis and nanomaterials for the prevention and treatment of NDDs.

## 1. Introduction

Annually, on a global level, an escalating trend of people affected by Neuro Degenerative Disorders (NDDs) [1]. NDDs are diseases related to neurons and neural circuits. NDDs include Alzheimer’s disease (AD) and Parkinson’s disorder (PD), glioblastoma (GBS), ischemia stroke (IS), and multiple sclerosis (MS) [2,3]. NDDs are always involved with the wasting of the cortex and hippocampus which causes abnormality in feeling and movement [4]. Currently, there is a huge gap in understanding and implementing a comprehensive treatment of such NDDs due to the blood–brain barrier (BBB) and less anatomical brain study [5]. To date, several investigations have shown the significant positive effect of conventional drugs on such NDDs [6] for instance Esposito and their group suspended the bromocriptine crystals with a combination of lipid tristearin/tricaprin and coated them with poloxamer-188 [7]. Further, the investigator obtained improved results with the nanosized bromocriptine for the treatment of PDs. In another study, in order to treat amyotrophic lateral sclerosis hydralazine was loaded on the mesoporous silica (SiO_2_) nanoparticles (NPs), and polyethylene glycol (PEG) was used to coat them. The combination of both the above-mentioned materials ameliorated the damage caused to both cell membranes and mitochondria. This process was induced by exposure to a normally lethal amount of acrolein in vitro [8]. For the treatment of MS, a group of investigators led by Basso formulated a nanosized fullerene derivative (water-soluble) (ABS 75) whose functionalization was carried out with an N-Methyl-D-aspartate receptor (NMDAR) antagonist [9]. From all the above investigations, it was found that there were two major drawbacks of conventional therapeutic drugs: (a) reduction in the effective drug dosage to the target sites and (b) inhibition in the growth of healthy cells [10,11]. This second factor becomes more crucial during the treatment of brain tumor or Glioblastoma (GBS)[12]. Hence, to overcome this issue there is a requirement for an emerging and efficient technology that could cross the BBB and deliver the drug effect for the treatment of NDDs [13]. Ferroptosis is a novel and emerging type of iron-dependent programmed cell death that is analyzed by the Fe-dependent lipid peroxidation (LiPr). This biological process involves a reduction in the activity of glutathione (GSH) peroxidase 4 (GPX4) and an accumulation of lipid peroxide (LiP) [14,15]. Ferroptosis is resultant of an imbalance between the production and degradation of Reactive Oxygen Species (ROS) [16]. A number of literature have shown that Fe and ferroptosis are associated with tumors and NDDs, like GBS, AD, and PD, as well as stroke, and are found very effective for the treatment of several cancers, and NDDs [17]. In all these disorders, ferroptosis is involved which mediates a cascade of molecular pathways. Ferroptosis activates several molecules that encourage various pathways for GBS, AD, PD, and stroke [18]. Previous investigations have shown that Fe^2+^ ions-based glutathione (GSH) activation results in the formation of ROS [19]. This particular pathway activates GPX4 molecules and ultimately leads to AD [20]. In the case of stroke, selenium activates GPX4 molecules, which activates tau (τ) protein where entire pathways are controlled by ferroptosis [21].

In the present review, an attempt has been made to emphasize the recent advancements in the ferroptosis-based treatment of several NDDs in association with the molecular mechanism of ferroptosis for NDDs. Here the authors have emphasized the recent progress in the nano-ferroptotic inducers for the therapy of ADs, PDs, and brain tumors (glioma and glioblastoma). Authors have also focused on various approaches of ferroptosis along with nanotechnology for the theragnostic approaches of NDDs. Emphasis was also given on the current and future challenges of nanoferroptotic-based therapy of NDDs and various clinical trials ongoing in this field.

## 2. Timeline of ferroptosis research

For the first time, ferroptosis was coined in 2012, after which numerous advances took place in this field. Various histories related to the ferroptosis research has been done in the last 20 years by various groups of investigators around the globe which is summarized below in Table 1. In these last 20 years, most of the molecules (including inducing ferroptosis regulators and ferroptosis inhibitors) along with their role in the ferroptosis pathway have been identified.

**Table 1.** Major milestones in ferroptosis research in last two deacdes.

Year	Milestones		References
	Molecules	Roles	
2003	Erastin	Mutant RAS selective compound	[22]
2007	Vitamin-E	Antioxidant	[22]
	VDAC2/3	Mitochondrial porins	[22]
	Mutated RAS	Oncogene	[22]
2008	TFRC	Iron transporter	[22]
	RSL3 RSL5	Mutant RAS selective compounds	[22]
	DFO	Iron chelator	[22]
2010	ML162 ML210	Mutant RAS selective compounds	[23–25]
2012	SLC7A11	Cystine/glutamate transporter. Coined the term ferroptosis	[22]
	Ferrostatin-1	Ferroptosis inhibitor	[22]
	Sulfasalazine	SLC7A11 inhibitor	[22]
2014	GPX4	Phospholipidhydroperoxidase	[22]
	Sorafenib	SLC7A11 inhibitor	[22]
	Liporoxststin-1	Ferroptosis inhibitor	[26]
	Zileuton	ALOX inhibitor	[27]
2015	SLC38A1	Glutamine transporter	[28–30]
	HSPB1	Heat shock protein	[31]
	TP53 (mutated tumor suppressor gene)	Transcription factor	[32]
	Artesunate	Antimalarial agent	[33]
	IKE	SLC7A11 inhibitor	[22]
2016	ACSL4	Lipid biosynthesis	[34]
	FIN56	GPX_4_ and coenzyme Q10 (CoQ10) inhibitor	[22]
	NEF2L2	Transcription factor	[35]
	NCOA4	Ferritinophagy	[28–30]
	ALOXs	Lipoxygenases	[22]
	FINO_2_	Inactivation of GPX_4_ & oxidation of Fe	[36]
	Statins	HMG-CoA reductase	[22]
2017	BH3-interacting domain death agonist (BID)	BCL2 family	[37]
	ZEB1	EMT-activator	[38]
	ITGA6-ITGB4	Cell adhesion	[39]
	Hemoglobin Hemin	Iron-containing protein	[40]
	Rosiglitazone	ACSL4 inhibitor	[41]
2018	BAP1	Epigenetic regulation	[42]
	NECTIN4	Cell clustering	[43]
	CTSB	Lysosomal cell death	[31]
	Withaferin A	Increase iron	[44]
	LOX-Block-1	ALOX inhibitor	[45]
2019	YAP1 NF2 WWTR1	Cell contact	[46]
	Apoptosis-inducing factor mitochondria-associated 2 (AIFM2)	CoQ10 production	[47]
	Cyst(e)inase	Cysteine depletion	[48]
	Ferroptocide	Thioredoxin inhibitor	[49]
	iFSP	AIFM2 inhibitor	[50]
2020	PEX10 PEX3	Peroxisome	[51]
	GCH1	BH_4_ production	[52]
	CHMP5 CHMP6	ESCRT-III membrane repair	[31]
	POR (p450 reductase)	Phospholipid peroxidation	[53]
	Zalcitabine	Antiretroviral agent	[31]
	Quercetin	Antioxidant agent	[54]
2023	Piezo1 & TRP channels	Cooperatively promote ferroptosis by facilitating cation flux	[55]

## 3. Ferroptosis and its characteristics

### 3.1. Ferroptosis

Ferroptosis [56] event is strongly linked to the Oxidative Stress (OS) response and metabolism of cystine as a governing form of nonapoptotic cell death [57]. From the initial investigation, it was assumed that ferroptosis differs from apoptosis at all three levels i.e. morphological, biochemical, and genetic. Moreover, investigators also suggested that the cells undergoing ferroptotic events generally show a necrosis-like morphological change [44]. A detailed observation by the investigators confirmed that necrosis, autophagy, shrinkage of mitochondria, and LiP deposition take place during ferroptosis which is similar to apoptosis. In ferroptosis cell shrinkage, chromatin agglutination, and other events do not take place [58–61]. Lipid oxidation in ferroptosis totally relies on the presence of Fe^2+^/Fe^3+^ ions inside the cell [62]. Additionally, when the intracellular oxidation–reduction is imbalanced, the Polyunsaturated Fatty Acids (PUFAs) in phospholipid (PL) molecules on the cellular membrane are oxidized and destroyed by LiP, which causes the rupture of the cellular membrane and cell death [63]. When the cellular glutathione-dependent antioxidant defense system gets inactivated there will be deposition of lipid ROS ultimately causing ferroptosis [64]. Cellular ferroptosis is characterized by abnormalities in intracellular lipid oxide metabolism, aberrant Fe ion-catalyzed metabolism, decreased antioxidant defenses, and an accumulation of lipid ROS [65], which leads to an unstable intracellular redox and causes cell death [64]. The three major, factors that lead to cell passage in ferroptosis are (a) an increase in free intracellular Fe, (b) a decrease in redox glutathione/GPX4/framework Xc, & (c) oxidation of layer PUFAs [66]. The ferroptosis further depends on various pathways like Xc- system /cysteine/GSH, dysfunction of varistor anion channels (VDACs) [67,68], p53 pathway [32,69,70], p62-Keap1-Nrf2 [71], ferroptosis suppressor protein 1 (FSP 1) as well as the transsulfuration pathway [72,73].

### 3.2. Important molecular players of ferroptosis

There are several important molecular players of ferroptosis which play a key role. These molecular players may act either as an inducer or inhibitor. Some of the important ones are described below in brief.

#### 3.2.1. SLC7A11

SLC7A11/xCT/system xc- are amino acid anti-transporters that are made up of two core components: SLC7A11 (light-chain subunit) and SLC3A2 (heavy-chain subunit). Both of these chains/components sustain the formation of an endogenous antioxidant GSH, through a series of reactions once it exchanges extracellular cystine for intracellular glutamic acid.

#### 3.2.2. GPX4

It acts as a PL hydroperoxidase which lowers the formation of PL hydroperoxide to the respective PL alcohol. The functions of GPX4 are governed by Se and GSH. Selenium could enhance the anti-ferroptosis features of GPX4 through the selenocysteine residue at 46 [74].

#### 3.2.3. AIFM2

The AIFM2 stands for ‘Apoptosis-inducing factor mitochondria-associated 2’ and is also called FSP1. It is a conventional inducer of apoptosis in the mitochondria and has been recently identified as a regulator of antioxidants in ferroptosis. N-myristoylation is needed for the translocation of AIFM2 from mitochondria to the cell membrane. After reaching the cell membrane, it catalyzes the regeneration of non-mitochondrial reduced CoQ10 by utilizing nicotinamide adenine dinucleotide phosphate (NADPH) which in turn traps the LiP in a GPX4-independent manner [75].

#### 3.2.4. CGL

CGL stands for cystathionine gamma-lyase which acts as the source of cysteine. The decomposition of the cystathionine (part of the transsulfuration pathway) is being carried out by CGL This pathway acts as a connecting link between methionine and GSH biosynthesis [76].

#### 3.2.5. NADPH

It is an important reducing agent, formed during the pentose phosphate pathway (PPP). It has an important role in limiting the damage of peroxidation caused by ferroptosis. It could be formed by the phosphorylation of NAD by NAD kinase (NADK). When the NADK silencing is done there is a reduction in the NADPH and erastin-, RSL3- and FIN56-induced ferroptosis, which increases [77].

#### 3.2.6. Aldosterone reductase family 1 (AKR1)

AKR1 is a family of aldo-keto reductase enzymes which has a significant contribution to steroid metabolism. Moreover, it includes both AKR1C and AKR1D subfamilies. In elastin-resistant tumor cells, there is an increased expression of AKR1C which inhibits ferroptosis by lowering the end products of LiP (AA/ AdA-PE-OOHs) to their respective nontoxic lipid-derived alcohols (AA/AdA-PE-OHs) [78].

#### 3.2.7. Peroxiredoxin (PRDXs)

It is a family of Se-independent GSH peroxidases that have a major role in suppressing ferroptosis. The OS is followed by the recruitment of PRDXs on the peroxidized cell membrane. Here PRDX6 minimizes and hydrolyses the oxidized sn-2 fatty acyl or the sn-2 ester (alkyl) bond of oxidized PLs. It prevents erastin- or RSL3-induced LOOH formation and ferroptosis via Ca^2+^-independent PLA2 activity [79,80].

#### 3.2.8. Thioredoxin

Its molecular weight is 12 kDa, which has an oxidoreductase activity. It is specifically located in the thioredoxin antioxidant system comprised of thioredoxin, thioredoxin reductase, and NADPH [81,82]. Ferroptocide is involved in the rapid induction of ferroptosis-like cell death in several cancerous cells by preventing the enzymatic action of thioredoxin [83]. The knockout of thioredoxin reductase 1 (TXNRD1) checks the ML210-induced ferroptosis in cancerous cells.

#### 3.2.9. GTP cyclohydrolase-1 (GCH1)

It is a rate-limiting enzyme of tetrahydrobiopterin (BH_4_) biosynthesis. BH_4_ is a major cofactor for numerous key enzymes that participate in the formation of dopamine and NO (neurotransmitters). GCH1-mediated BH_4_ formation leads to lipid remodeling and inhibits ferroptosis by selectively preventing two polyunsaturated fatty acyl tails from utilizing PLs. The deficiency of BH_4_ could play a significant role in the pathogenesis of ferroptosis-based disorders.

### 3.3. Different mechanisms of ferroptosis

**Figure 1.**
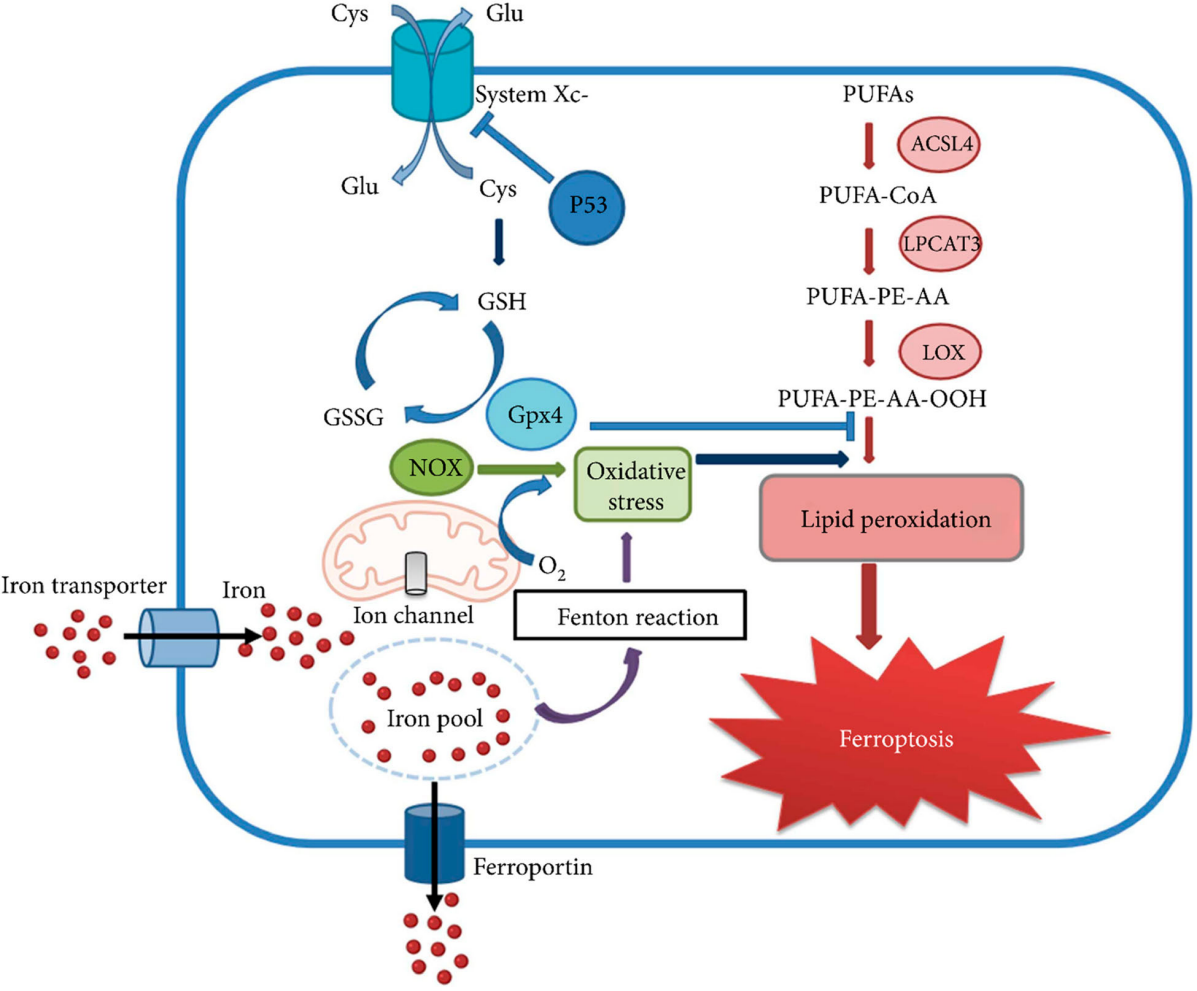
Mechanism pathways of ferroptosis adapted from [16].

Several researchers have detailed the various ferroptosis pathway mechanisms, the most simplified of which is explained in the present study. Ferroptosis is induced either by amino acid metabolism, Fe metabolism, or LiPr [63] which are explained in the following discussion. Figure 1 exhibits the mechanisms involved in the ferroptosis pathway.

#### 3.3.1. Iron metabolism-based ferroptosis pathway

Iron being an essential element is required for numerous cellular functions like deoxyribonucleic acid (DNA) synthesis, O_2_ transport, cellular respiration, and biosynthesis of neurotransmitters in the nervous system [84,85]. The homeostasis of iron plays a major role in the survival and formation of normal cells [86]. On the contrary, its deficiency leads to anemia [87]. Ferroptosis is marked by the deposition of iron while an excess of iron will lead to an elevated risk of cancer [88]. A significant number of studies showed that there are several Iron Regulatory Proteins (IRP) (IRP1 and IRP2) that control the cellular iron metabolism by posttranscriptional control. Both types of s could regulate the iron metabolism genes transferrin receptor (TFRC) and ferritin heavy chain 1 (FTH1) under normal physiological environments to maintain the stability of unstable iron pools, where the LIPs, are made up of a lesser quantity of free Fe^2+^ [89]. Iron is mainly available in either Fe^2+^ (ferrous) or Fe^3+^ (ferric), while the Fe that circulates in the blood is mainly Fe^3+^ after binding to transferrin (TF). Firstly, Fe^3+^ ions are brought inside the cell by various iron transporter proteins i.e. transferrin receptor 1 (TFR1), and lead to the formation of an intracellular iron pool. From this iron pool, some of the iron is discharged out from the cell as ferroproteins while the remaining iron pool is involved in the Fenton reaction and leads to OS. During this Fenton reaction, there is a release of O_2_ from the mitochondria. O_2_ along with NO_X_ causes OS inside the cell followed by LiPr which ultimately leads to ferroptosis [90]. The detailed events involved in iron-mediated ferroptosis are discussed below. Fe-mediated ferroptosis begins with the entry of free Fe^3+^ ions into the cell via a TFR 1 (cell membrane protein). These Fe^3+^ ions accumulate inside the nucleosome of the cell which is further reduced to Fe^2+^ with the help of nucleosome iron reductase-prostrate hexame transmembrane epithelial antigen 3 (STEAP 3). The reduced form of iron gets transported from the endosome to the cytoplasm facilitated by divalent metal transporter 1 (DMT 1). In general, Fe^2+^ ions get deposited into the ferritocyte stock protein complex, which is made up of FTH 1 and ferritin light chain (FTL), in order to maintain the balance of unstable pools of iron and inhibit the generation of ROS [91]. Some fraction of Fe^2+^ ions get exported to the extracellular space with the help of a ferritin FPN 1 (membrane protein). Further, if there is any failure in the uptake, transport, storage, and use of intracellular iron, then there will be excess Fe^2+^ ions accumulation inside the cell. This will lead to the initiation of the Fenton reaction, ultimately leading to the generation of (°OH) and ROS. The ROS generated in the previous step in turn modifies and interferes with the biological molecules of the cell (proteins, lipids, and DNA). Moreover, there is an occurrence of sequential peroxidation reactions with PUFAs on the cell membrane which leads to the generation of LiP [92]. Due to the formation of LiP, there is destruction in the cell morphology ultimately leading to cell ferroptosis. The deposition of iron in the cell is especially due to the following barriers i.e. membrane iron transporter (FPN), TFR 1, and DMT 1 [93]. Due to all these barriers, there is a loss of control over iron transport. Alternative to this, nuclear receptor coactivator 4 (NCOA4)-based degradation of ferritin phagocytosis pathway may get initiated which may result in the enhanced storage of iron [94]. Further, there is a Fenton reaction/mitochondrial damage/lipoxygenase (LOX) function which may ultimately lead to enhanced iron accumulation in the active iron pool. Finally, due to all the above events, there is an increased ROS which eventually results in ferroptosis.

#### 3.3.2. PUFAs-based ferroptosis pathway

PUFAs-mediated pathway is another mechanism, where PUFAs get converted into PUF-CoA in the presence of ACSL4. Further, PUF-CoA gets converted into polyunsaturated fatty acid (PUF)-phosphatidylethanolamine-(PE) arachidonic acid (AA) [PUFA-PE-AA] in the presence of lysophosphatidylcholine acyltransferase 3 (LPCAT3). PEs having AA are one of the key phospholipids that induce cellular ferroptosis. Further, PUFA-PE-AA gets converted into polyunsaturated fatty acid (PUFA)-phosphatidylethanolamine-(PE)[PUFA-PE-AA-OOH] in the presence of lipoxygenase (LOX). At this point, the molecules get affected by the OS and there is lipid oxidation which leads to ferroptosis [95]. The detailed mechanism of these pathways is described below in detail. The pathways start with the formation of ROS (OH) which triggers the LiPr to form lipid radicals and lipid peroxy radicals [96]. These lipid radicals further react with the PUFAs to form LiP which ultimately leads to ferroptosis. Here in total, these pathways are involved in the iron participation in the accumulation of ROS. ROS further interacts with the PUFAs in the lipid membrane which induces LiPr, which in turn triggers intracellular ferroptosis. The dyalenyl H atoms of PUFAs react readily with the ROS leading to LiPr and ultimately leading to the death of cellular iron [21]. Further, phospholipids (PEs) having AA induces cellular ferroptosis. In the next step, there is an enhanced ferroptosis which is achieved by supplementing with AA/other PUFAs and inhibiting the LPCAT 3 and Acyl-CoA synthetase long-chain family member 4 (ACSL4) activity. In order to generate the ferroptosis signals there is a requirement for the generation of PUFA and coenzyme A (CoA) derivatives followed by their binding with the PLs [97]. These could be the potential targets for the treatment of disorders involved with ferroptosis.

#### 3.3.3. Cystine/glutamic acid-based ferroptosis pathway

**Figure 2.**
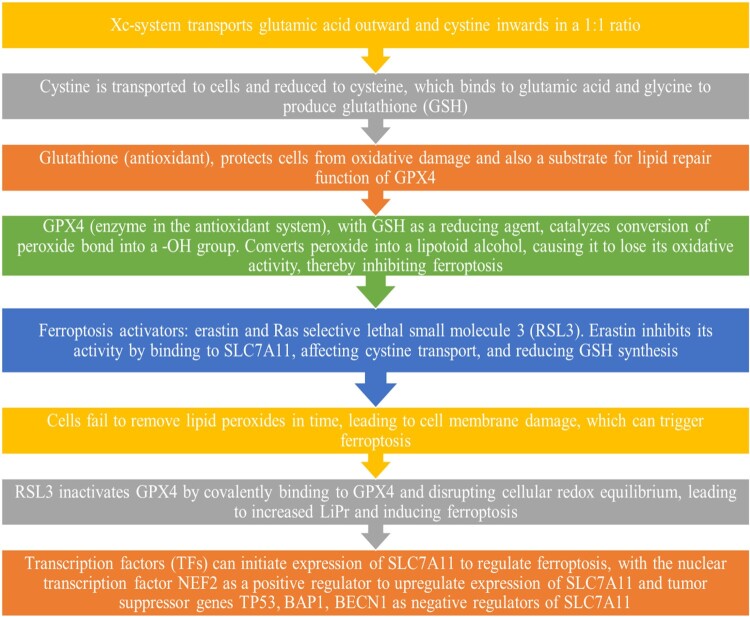
Cystine/glutamic acid-mediated ferroptosis pathway.

**Figure 3.**
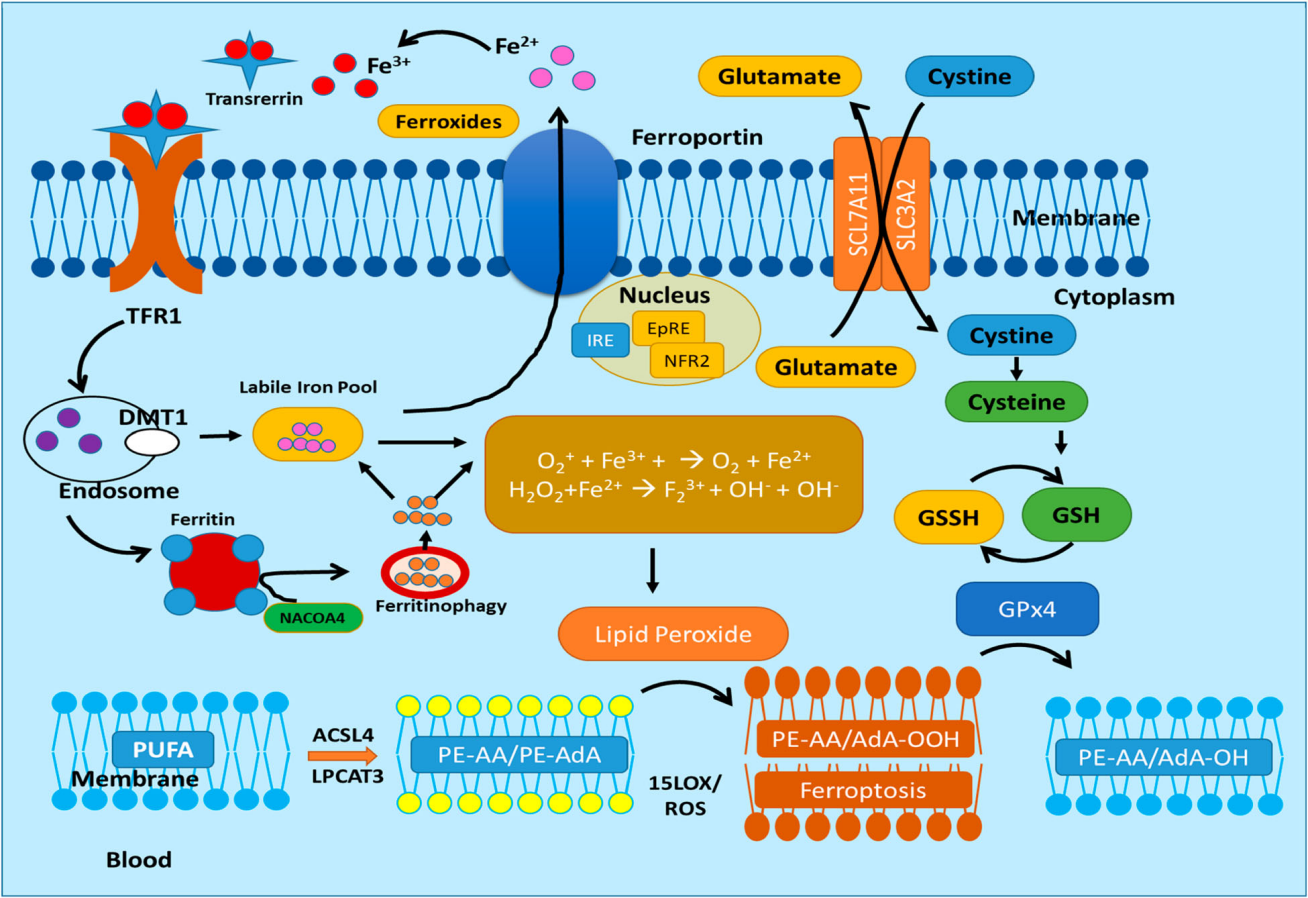
Ferroptosis mechanism pathways adapted from [14].

The third mechanism is the cystine/glutamic acid-mediated pathway where cystine/glutamic acid metabolism contributes a significant role in ferroptosis [98]. In this particular pathway, there is an Xc-system, which is an amino acid antiporter that mainly facilitates the exchange of extracellular l-cystine and intracellular l-glutamic acid across the plasma membrane of the cell [99]. The Xc system is comprised of light-chain solute carrier family 7 members 11(SLC7A11) and heavy-chain solute carrier family 3 members 2 **(**SLC3A2) which are attached by disulfide bonds. It transports the glutamic acid outward of the cell whereas cysteine is transported inward to the cell by maintaining a 1:1 ratio (glutamic acid: cysteine). The glutamate further gets converted into glutathione (GSH), which reversibly gets oxidized and converted into oxidized GSH (GSSG). The glutathione-dependent peroxidase (GPX4) molecule gets activated which further joins the PUFAs-mediated pathway and Fe-mediated pathway, leading to LiPr and causing ferroptosis [100]. Figure 2 showed a typical cystine/glutamic acid-mediated ferroptosis pathway [101]. Figure 3 depicts a combined ferroptosis pathway mediated by cystine/glutamic acid, LiPr oxidation, and iron metabolism.

## 4. Ferroptosis inducers and inhibitors and other causes of ferroptosis

A ferroptotic event in a cell is controlled by the various types of biomolecules and inorganic materials which may act either as an inducer or inhibitor for ferroptosis [102]. There are several molecules in a cell that could trigger/induce the ferroptosis event for instance erastin, FINO2, FAC, statins, FIN56 etc. Besides this, there are several molecules that may affect the ferroptotic event in a cell by inhibiting i.e. DFO, selenium, dopamine CoQ10, etc. [103]. Ferroptosis regulators can be broadly divided into two categories i.e. inhibiting ferroptosis and inducing ferroptosis [97]. Inhibiting ferroptosis regulators are mainly Se, CoQ10, NRF2, Fanconi anemia group D2 protein (FANCD2), and NFE2L2 whereas inducing ferroptosis includes NADPH, p53, and BECN1 (Beclin 1) [97]. All these regulators have different roles either in inhibiting or inducing the ferroptosis. Both inducers and inhibitors of ferroptosis have different mechanisms on the ferroptotic event [104], which are briefly discussed below and given in Table 2.

**Table 2.** Inhibitors and inducers of ferroptosis.

Role	Mode of action	Small molecules	Nanoparticles	References
Inducer	Fe homeostasis	FAC	Fe-based NMs like IONPs, Fe-organic NPs, FePt	[116]
	NRF2 inhibition	Trigonelline, brusatol	–	[117]
	LiPr	FINO2	WS_2_, Fe-organic NP, FePt & MoS_2_	[36]
	Inhibition system xc −	Sulfasalazine; glutamate, erastin, PE, sorafenib, IKE erastin analogs	–	[118]
	GSH depletion	Cystine/cysteine deprivation, acetaminophen, cisplatin, BSO, DPI2, cysteinase	Zinc oxide NPs	[119]
	Suppression GPX4	(1S,3R)-RSL3; ML162, FIN56; DPI family members	Fe-free NMs, (WS_2_, MoS_2_ & Copper NPs)	[120]
	CoQ10 biosynthesis inhibition	Statins	–	[121]
Inhibitor	System xc− activation	β-mercaptoethanol, Cycloheximide	–	[122]
	Fe chelators	DFX, CPX, DFO, DFP	–	[116]
	Selenoprotein increment	Se	–	[116]
	Reduction of LiPr	Vit-E, BHT, BHA, Fer-1, AA-861, zileuton; vildagliptin, alogliptin, trolox, tocotrienols, Lip-1; CoQ10, idebenone; XJB-5-131; deferoxamine, cyclipirox, deferiprone; CDC, baicalein, PD-146176 and linagliptin	CPS	[123]
	GPX4 upgradation	Dopamine	–	[124]

Selenium (a ferroptosis inhibitor) is an essential micronutrient that maintains the GPX4 activity, which in turn activates the abundance and activity of GPX4 [105,106].

There is a synergistic activation of the transcription factor AP-2 gamma (TFAP2c) and Sp1 transcription factor (Sp1), by preventing ferroptosis to a certain extent for the protection of neurons [21,107].

Another inhibiting ferroptosis regulator is CoQ10, whose concentration in the cells is lowered by ferroptosis suppressor protein 1 (FSP 1) [108], to prevent LiPr and inhibit ferroptosis. Hence FSP 1 could prove to be an important target for the treatment of similar disorders [109,110].

NRF2 has a significant role in the upregulation of the expression of gene clusters engaged in the iron and ROS metabolism NAD(P)H quinone oxidoreductase 1 (NQO1), FTH1 through the p62-Keap1-NRF2 pathway and heme oxygenase 1 (HO1) [111].

Another important ferroptosis inhibitor is FANCD2 which helps in the regulation of expression of protein through both transcription-dependent and nondependent mechanisms.

NFE2L2 minimizes oxidative damage (OD) during ferroptosis wherein NFE2L2 regulates the expression of related genes (associated with the metabolism of Fe and GSH, and the anti-ROS process) through transactivation plays an important role in the regulation of the expression of related genes by transactivation to restrict OD during ferroptosis [97]. Several literary works suggest that the NFE2L2 signaling pathway is a very crucial defense method against ferroptosis [112].

Among inducers of ferroptosis, one of the important molecules is NADPH which controls or mediates the circulation of the GSH-GPX4 antioxidant system [113]. The heavy consumption of GSH-GPX4 will restrict the antioxidant activity of GSH-GPX4. If NADPH is consumed higher than this will control the activity of GSH-GPX4 and can trigger ferroptosis [114].

The inhibition of cysteine uptake by downregulating the expression of SLC7A 11 (Xc- system component) is controlled by the p53 regulator.

BECN1 inhibits the activity of the Xc- system and blocks cysteine output, which eventually results in the occurrence of cellular ferroptosis [115].

## 5. Hallmarks features of ferroptosis

**Figure 4.**
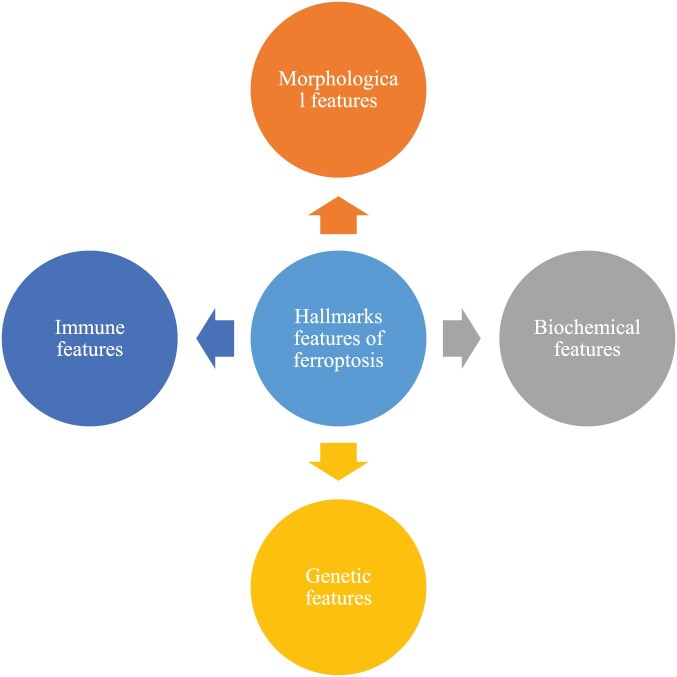
Hallmarks features of ferroptosis.

Ferroptosis in a cell is marked by its several hallmark features which are broadly classified into four groups namely morphological features, biochemical, genetic features, and immune features. Figure 4 shows the hallmarks features of ferroptosis.

### 5.1. Morphological features

In ferroptosis the morphological features are marked by necrosis-like changes in the shape and size of various cellular organelles, loss of integrity of plasma membrane, swelling of cytoplasmic organelles and cytoplasm (oncosis) [49], condensation of chromatin, cells detachment and rounding up, and increased autophagosomes [125]. It is the tendency of a ferroptotic cell to spread quickly in the adjacent cell [126]. The detailed study at the ultrastructural level of a cell undergoing ferroptosis revealed that during ferroptosis cell shows mitochondrial abnormalities i.e. swelling/condensation, density of membrane increases, lowering or absence of crista, and rupturing of outer membrane [127]. Some recent investigations have exhibited that mitochondria-based ROS generation, DNA stress, and metabolic reprogramming are needed for LiPr and induction of ferroptosis [127,128].

### 5.2. Biochemical features

From the various literature, it has been confirmed that ferroptosis is a ROS-dependent apoptosis that is present with two basic features i.e. iron accumulation and LiPr [96].

#### 5.2.1. Iron accumulation

Ferroptosis activators like erastin or RSL3 cease the antioxidant system once they enhance the deposition of iron inside the cell [129]. The iron deposited over here will directly produce excessive ROS via the Fenton reaction, leading to an increase in OD [130]. Moreover, the activity of enzymes namely LOX or Egg-laying defective nine (EGLN) prolyl hydroxylases is increased by iron accumulation, where the roles of the enzymes are LiPr and oxygen homeostasis respectively [49,94,131,132]. The sensitivity of ferroptosis depends on the dynamics between systemic and local cellular regulation of Fe. Further, ferroptotic cell death is effectively inhibited either by targeting genes associated with iron overload or due to the use of iron-chelating agents [133].

#### 5.2.2. Lipid peroxidation (LiPr)

LiPr is a free radical-propelled reaction that specifically affects the unsaturated fatty acids in the cell membrane [134]. The various LiPr products are initial lipid hydroperoxides (LOOHs), and subsequent reactive aldehydes [malondialdehyde (MDA) and 4-hydroxynonenal (4HNE)], whose concentration increases during the ferroptosis [135]. Here there is an involvement of mainly three types of fatty acids namely: saturated fatty acids, monounsaturated fatty acids (MUFAs,) and PUFAs [136,137]. During the ferroptotic event, various lipids of the cell membrane like phosphatidylcholine, phosphatidylethanolamine (PE), and cardiolipin get oxidized but peroxidation of PUFAs in phospholipids by LOX is highly important for ferroptosis. The peroxidation of cardiolipin has not been observed yet in ferroptosis [63].

### 5.3. Genetic features

Several investigations have revealed that the ferroptotic event is marked by the overexpression of certain genes/proteins, for instance, prostaglandinendoperoxide synthase 2 (PTGS2/COX2)[required for prostaglandin biosynthesis] [138]. Another such enzyme is Acyl-CoA synthetase long-chain family member 4 (ACSL4) which plays an important role in the metabolism of fatty acid [139]. It is considered an important biomarker and driver of ferroptosis as the upregulation of ACSL4 enhances the PUFA content in phospholipids. These enhanced PUFA contents are prone to oxidation reactions directing to ferroptosis [140]. Genes having an important role in the antioxidant defense get activated during ferroptosis [e.g. GSH & CoQ10 system, and nuclear factor erythroid 2-like 2 (NFE2L2/NRF2) transcription pathway] along with membrane repair (e.g. the endosomal sorting complexes required for transport (ESCRT)-III pathway34), that lowers the damage of the cellular membrane during ferroptosis [64]. Hence based on the balance of the injury and anti-injury responses, a cell decides to live or to die in response to the stimulus of ferroptosis.

### 5.4. Immune features

Ferroptosis has two significant immunological effects on the cell, one is the death of leukocyte types and the corresponding loss of immune activity, for instance, LiPr induces ferroptosis in T cells and favors viral or parasitic diseases [141]. Secondly, when non-leukocytic cells are affected by ferroptosis, it becomes very important how the dying cells or forming corpses are handled by the immune system [142]. This is more important as the death of different cells may give rise to different immune and inflammatory responses through releasing and activating different damage-associated molecular pattern (DAMP) signals. During ferroptosis, the inflamed cells die which are associated with the DAMPs or LiPr at the time of tissue injury or tumor therapy [143]. The 4-HNE is a pro-inflammatory mediator that is formed during the LiPr. This LiPr product is engaged in the activation of the nuclear factor-κB (NF-κB) pathway, in aging and chronic diseases [144]. Another one is high mobility group box 1 (HMGB1), which is a prototypical DAMP that plays an important role in cell death. HMGB1 is discharged by the ferroptotic cells which in turn triggers an inflammatory response in peripheral macrophages by a specific pathway [145]. In order to treat inflammatory diseases that arise from ferroptotic damage, one has to target the lipid metabolism-related DAMP signaling which could prove to be a promising strategy [94].

## 6. Detection procedures of ferroptosis

### 6.1. Biomarkers associated with ferroptosis

**Figure 5.**
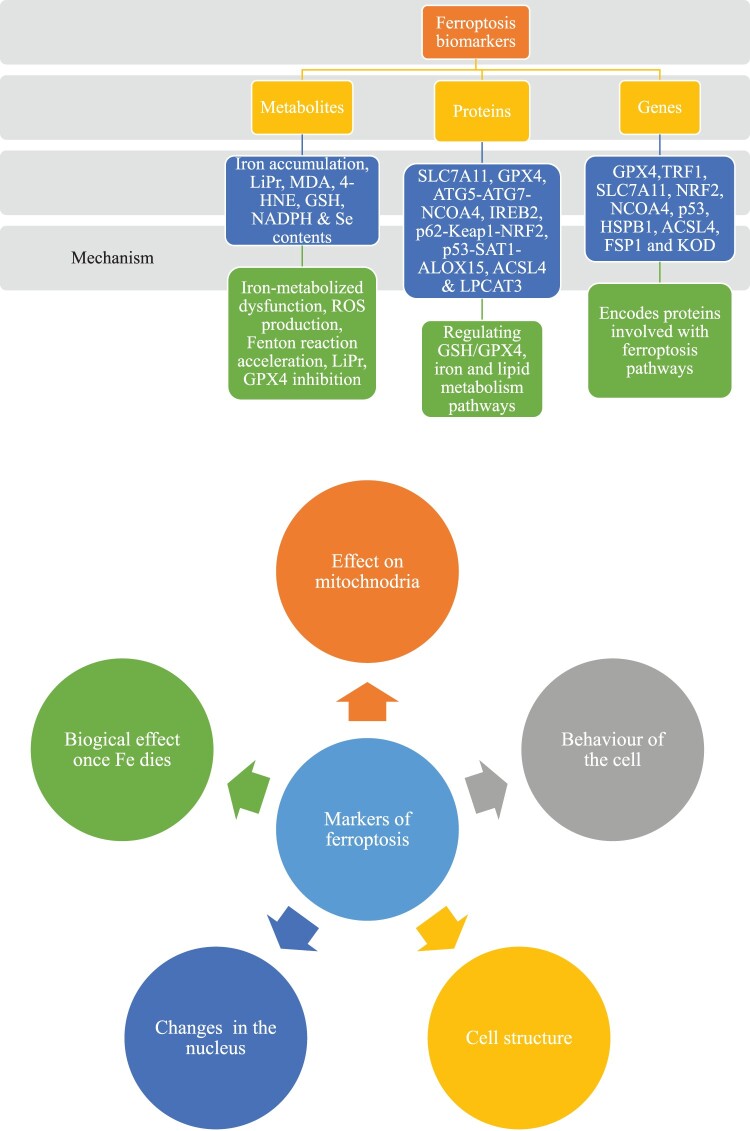
Various markers of ferroptosis.

There are several ferroptosis markers that can be used to confirm the ferroptotic event in the cell [146]. A biomarker in ferroptosis could be mainly categorized into three types on the basis of their biological nature. A biomarker in ferroptosis could be either metabolites, proteins, or genes [147] which is shown in Figure 5. A ferroptotic event is marked by several cellular behaviors for instance, the effect on the mitochondria, behavior of the cell, morphology of the cell, and its nucleus, and the biological effect on the cell once the iron dies [146,148] (Figure 5), which discussed below in detail.

During the ferroptotic event, mitochondria act as an important marker as it goes various changes like there is a decrease in the size and numbers of mitochondria, mitochondrial atrophy, decrease/disappearance in the mitochondrial spine [149]. Moreover, some of the ferroptotic events in mitochondria could also be marked by the increase in the mitochondrial membrane density which could be due to the dysfunction of the VDACs and changes in the mitochondrial membrane fluidity [150]. Another important cellular ferroptotic marker is the behavior of the cell, which could be marked by the shedding and aggregation of the cells and a drastic rise in the intracellular autophagosomes [151]. Cellular ferroptotic events could also be marked by the cell structure for instance rupture of the cell membrane and the formation of a bubble [152]. Several investigators have also marked the cellular ferroptotic event by visualization of the nucleus features for instance there will be devoid of chromatin agglutination, comparatively smaller intracellular mitochondria, rupturing of the outer membrane of mitochondria, and enhanced bilayer membrane density [152].

### 6.2. Procedures applied for the assessment of ferroptosis

#### 6.2.1. Microscopy imaging

Ferroptosis involves several proteins, molecules, and genes that bring about several changes at the molecular level [151], which can be observed by using microscopic techniques like transmission electron microscopy (TEM) and confocal microscopy [153]. Moreover, these microscopic techniques could provide a detailed structure at the subcellular level. Earlier Zhang and coworkers observed the mitochondrial shrinkage with fused cristae of mitochondria in a ZnO NPs-treated Human umbilical vein endothelial cells (HUVEC) under TEM.

#### 6.2.2. Mass spectrophotometry

The ferroptosis involves LiPr, a pathway that generates LiPr products which can be examined by mass spectrometry. The MS mainly provides mass to charge ratio (m/z), of the lipid particles and generates mass spectra that can provide data about molecular mass, elemental components, and chemical structure of lipids [154]. Previously, Kagan and their team utilized liquid chromatography coupled with MS, in order to detect the structure of LOOHs in ferroptosis [155]. A team led by Isabel applied matrix-assisted laser desorption/ionization (MALDI) based MS for the investigation of the role of oxidized PUFAs in ferroptosis [156].

#### 6.2.3. Western blotting

Ferroptosis involves several biological protein molecules like SLC7A11, GPX4, TRF2, etc. which have been identified by western blotting. Previously a team led by Eleftheriadis examined the expression of these protein molecules involved in a ferroptotic event of a cell [157]. Wang and their team have used this method to reveal the activity of glycyrrhizin on ferroptosis in acute hepatitis failure [158]. Zhou and their coworkers applied western blotting and found that there was a continuous decrease in the proteins (GPX4 and SOD2) in the ferroptosis [159].

#### 6.2.4. Genetic analysis

As ferroptosis is controlled by several genes, the investigation of ferroptosis could be done by either genetic analysis or by gene mutagenesis [160]. The former technique applies RNA interference screening and genome screening for the identification of relevant genes [161]. A team led by Gao applied RNAi screening to a wider range of investigations of ferroptotic cells. By applying similar techniques, ferroptosis genes and some uncorroborated genes were also found to be involved with ferroptosis [162]. A team led by Cao reported the utilization of genome-wide human haploid cell genetic screening methods to investigate the genes that are involved in the intracellular regulation of GSH abundance and their importance in regulating ferroptosis [163].

#### 6.2.5. Other methods

As ferroptosis involves Fe the ferroptotic event in a cell can be measured by the analysis of Fe ion by the inductively coupled plasma (ICP)-MS (ICP-MS) [164]. ICPMS are highly accurate techniques for the quantification of Fe content in biological systems. Previously Pepper and their coworkers have used ICP-MS for the estimation of Fe^3+^ ions in an organic phase in order to distinguish between Fe^3+^ and Fe^2+^ ions in the biological systems [165]. Several investigators have also used fluoresce spectrophotometry for differentiating ferrous and ferric ions in biological systems by applying fluorescent probes. Fluorescent probes specifically chelate Fe^2+^ (nanthroline and ferrozine) and Fe^3+^ (Rhodamine B hydrazonespirolactam) with an alternation in their spectra [166]. The ferrous and ferric phases of iron could easily be distinguished by using Mössbauer spectroscopy and Absorption near edge spectroscopy (XANES), but these two techniques rarely have been used so far.

## 7. Mechanism of nanomaterial-induced ferroptosis

The NMs-induced ferroptosis could exhibit significant inferences in nanomedicines and nanosafety. From the various investigations, it has been observed that the NMs-induced ferroptosis has the equivalent classical features as small molecule inducers e.g. inhibition of GPX4, Fe overloading, and LiPr. It has been observed that the initial molecular reactions in the NMs-based induced ferroptosis pathway are completely different. Zheng and their team have proposed three ferroptosis pathways on the basis of reported ferroptosis signals induced by NMs. These pathways are membrane impairment, lysosomal dysfunction, and mitochondrial damage [167].

### 7.1. Membrane impairment

The free access of exogenous NPs is controlled by the cellular plasma membrane [168]. Some of the NMs like fumed silica and graphene oxide were strongly associated with the plasma membrane and least in the lysosomes [169]. XC^-^ system and TRF1 are valuable upstream proteins in Fe metabolism therefore the binding of NMs on the plasma membrane may alter the biological activity of these proteins [129]. A team led by Herbison found that Co(II)Tf and Mn(II)Tf could upregulate the TFR1 and reduce ferritin that could affect iron homeostasis. Mn^2+^ ions could utilize the same imported (DMT1) with ferric ion which may have a competition and may affect the uptake of iron homeostasis [170].

### 7.2. Lysosomal dysfunction

Ferroptosis is closely associated with the lysosomal dysfunction. A surplus amount of redox-active iron is being deposited in the lysosome [116]. The lysosome may undergo undesirable reactions with endocytic NMs, out of which some of the NMs may get transformed into the acidic and enzymatic organelle to elicit impairment of lysosomes by redox reactions, denaturation of biomolecules presents in lysosomes, and physical interactions [116]. Zheng and coworkers exhibited that the dysfunction of lysosomes by MoS_2_ and WS_2_ nanosheets could result in the discharge of the Fe^2+^ ions in the cytoplasm. These free Fe^2+^ ions triggered the formation of ROS by the Fenton reaction and also induced LiPr which leads to ferroptosis. When both the nanosheets were modified by the Na_2_S or methanol ameliorated the impairment of lysosome and minimized the secretion of Fe^2+^ ion in the cytoplasm, which played a major role in the improvement of cell viability [171,172]. A team led by Wang utilized amine-modified polystyrene NPs, which resulted in the release of lysosomal enzymes and iron to activate ferroptosis [173].

### 7.3. Mitochondrial damage

**Figure 6.**
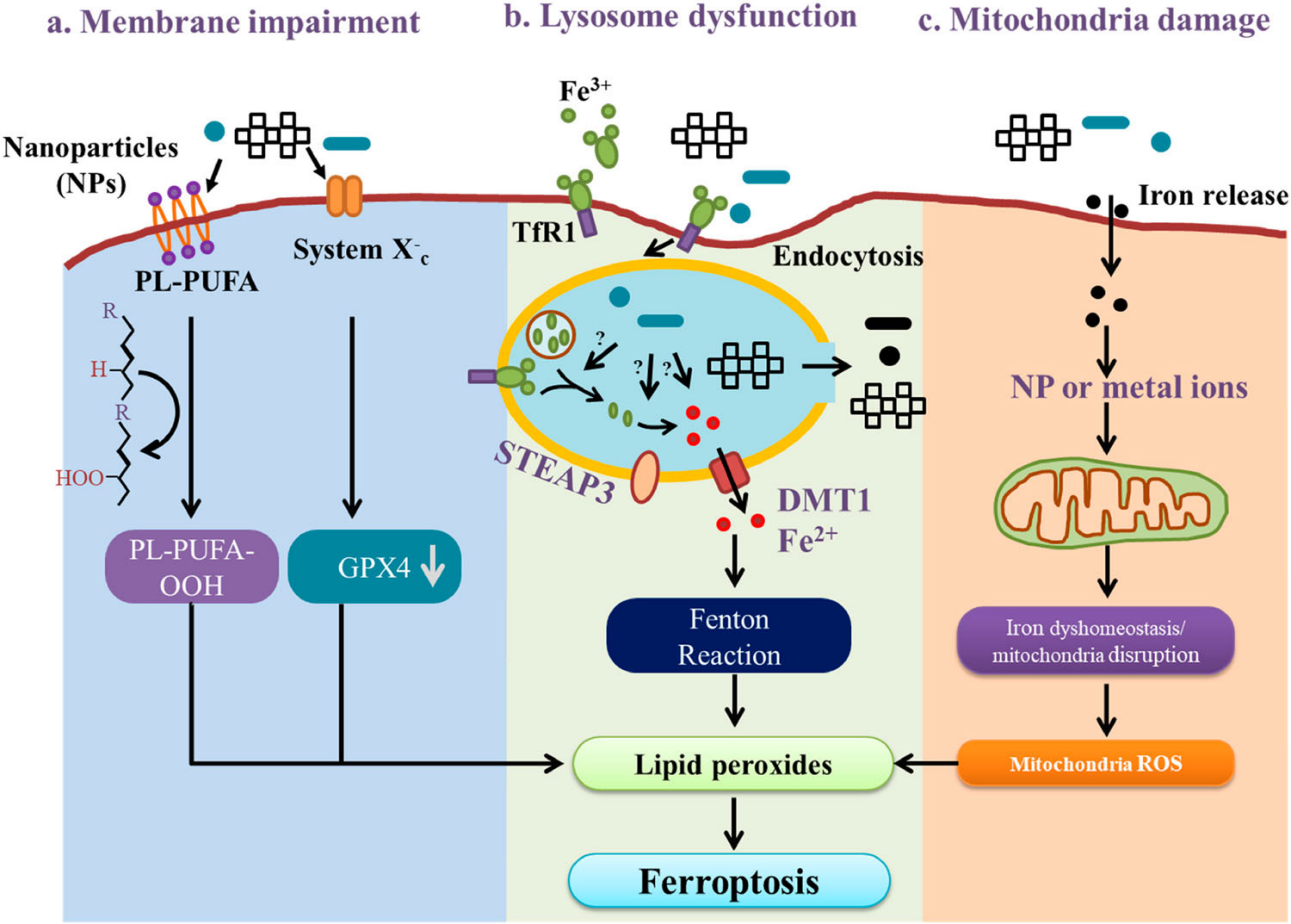
Mechanisms of NPs-induced ferroptosis. (a) Membrane impairment induced by NPs involving LiPr and inactivation of system xc-; (b) lysosome dysfunction induced by NPs including disruption of lysosomal membrane, alteration of acidic environment, modification of STEAP3 and DMT1 activities; and (c) mitochondrial damage induced by NPs including destruction of mitochondrial morphology and dysregulation of the mitochondrial antioxidant defense as well as iron dyshomeostasis.

Mitochondria is one of the most important subcellular organelles which adds a role in apoptosis, autophagy, and ferroptosis [174]. Numerous investigations have exhibited a change in the shape and size of the mitochondria in the ferroptotic cells that were induced by the NMs [175]. Zhang and coworkers have developed a FePt@MoS_2_ nanocomposites (ferroptosis agent) that could discharge 30% ferrous ion within 3 days in the tumor microenvironment for inducing the ferroptosis by accelerating the Fenton reactions [176]. A team led by Huang utilized zero-valent iron (ZVI) NPs to control the ferroptosis where the oxidative conversion of ZVI to ferrous ion assisted the Fenton reaction for inducing the mitochondrial LiPr and MDA formation [177]. Zhang and their colleagues showed that Zn^2+^ dissolved from zinc oxide NPs could upregulate the mitochondrial VDAC proteins [178]. All these three pathways are shown in Figure 6.

## 8. Neurological diseases associated with signaling pathways of ferroptosis

Ferroptosis-based approaches for treating various neurological-related diseases are mainly due to the reason that the iron ions participate in the various cancerous cell cycles by altering the DNA replication and repair pathway [179]. From the investigations, it has been revealed that the neoplastic cells have increased iron concentration than the non-cancerous cells [180]. So, there is a scope for the iron-based signaling pathway to inhibit cancer growth [181,182]. Several investigators found that in the unavailability of antioxidant protection, efficient killing of the cancerous cells was attained by inducing Fe-dependent OD via the ferroptotic pathway [182]. To date, numerous ferroptotic inducers both in micron and nanosized have been synthesized to upgrade the currently ineffective anti-tumor approaches. The application of novel inducers of ferroptosis with targeted nanocarriers has several advantages improved drug stability, prolonged plasma half-life, facilitated cellular internalization, and enhanced accumulation at the tumor sites [183]. All these factors help in the eradication of cancerous cells. In one of the investigations carried out by Ma and coworkers, it was found that the IONPs were used as a carrier for cisplatin (IV) for enhanced anti-tumor activity and reduced systemic toxicity [184].

### 8.1. Ferroptosis in general diseases

Several investigators have shown the role of ferroptosis in the various organ-related disorders in the body [15]. Zhang and their team reported the involvement of ferroptosis in the various disorders associated with acute kidney injury, cancer, hepatic fibrosis, PDs, and ADs [18]. More especially, it is involved in all types of cancer of the liver, gastrointestinal tract, kidney, and lungs [185,186]. In addition, it has an important role in CNS-related disorders [96,187,188]. Since OS and Fe accumulation are the trademark pathological characters of NDDs, the importance of ferroptosis in NDDs has been investigated a lot [189].

### 8.2. NDDs associated with ferroptosis

Neurological disease is considered to occur when the central nervous system gets damaged which leads to an increase in the ROS protein nitration [190]. The most reliable approach to reduce the progression of NDDs is to enrich the body with antioxidants which will stop the overproduction of ROS [191]. The majority of NDDs have a common pathological mechanism like damaged protein, quality control, and degradation pathway, dysfunctional stress granules of mitochondria, and incompatible innate immune responses (ImR) [192,193]. Moreover, NDDs also exhibit unique pathologies and clinical features in different parts of the brain [194,195]. Several investigations have shown that ferroptosis is present with several NDDs (ADs, PD, Huntington's disease), strokes, and various types of cancer. Ou and their team reported that NMs like low-density lipoprotein–docosahexaenoic acid NPs could specifically trigger ferroptosis in liver cancer cells by LiPr, lowering of GSH, and inactivation of GPX4 [196].

**Figure 7.**
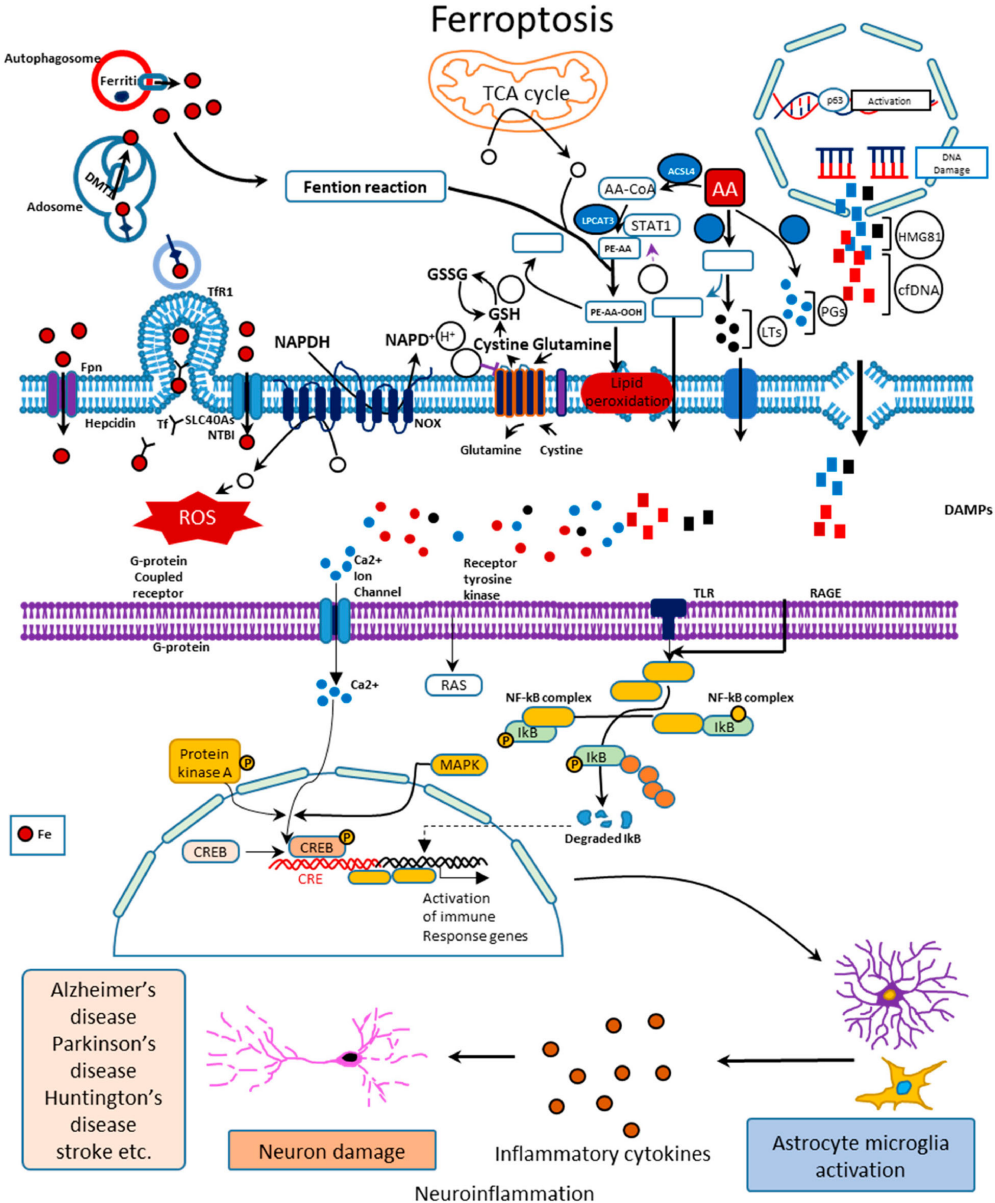
Putative pathway for ferroptosis participates in neuroinflammation to NDDs reprinted from [16].

Figure 7 shows a putative pathway for ferroptosis that takes part in neuroinflammation to neurological diseases [16]. Here DAMP molecules [ROS, cfDNA, ITs, HMGB1, and PGs] generated during the ferroptosis events, activate glial cells (AGC) by activating neuroimmune pathways. Further, these AGCs produce a series of inflammatory factors that add to neural impairment and a series of NDDs (Huntington’s disease, ADs, PDs, GBS, and strokes).

#### 8.2.1. Alzheimer’s disease (AD)

Alzheimer’s disease is a chronic NDDs with prolonged preclinical stages along with an average clinical time of 8–10 years [197,198]. Every year around 30 million people are affected by ADs and predicted to increase to 106 million by 2050 [199–201]. In this disease, there is mainly deposition of amyloid beta (Aβ) plaques present outside the cell and neurofibrillary tangles (NFT) in the brain [202]. Several literary works have evidenced that AD occurs due to a complex synergy, for instance, genetic susceptibility [203,204], aging [205], environment [206], occupation [207], and overexposure to metals [208,209]. Breijyeh and their team presented a detailed overview of the causes and treatment of AD [208]. The synaptic function is attenuated by the pathogenic forms of Aβ and τ which result in activating an order of events that results in the death of neurons [210,211]. Presently the exact pathogenesis events of AD is not known thoroughly, so the only option to minimize the risk of this disease is clearing Aβ and τ in NFT in neurons [212]. Moreover, the risk of AD could be reduced by either preventing or interfering with Aβ and τ aggregates which will minimize membrane damage, cell apoptosis, intracellular microtubule impairment, and ROS generation [213,214].

Several investigations have shown that the ferroptotic events are also present in the AD out of which the most prominent are excess accumulation of iron, increased LiP, and ROS. All these events are associated with typical clinical features, for instance at the time of excess iron accumulation there is a higher concentration of iron in the brain of patients suffering from AD [102]. Another important clinical feature is brain atrophy coincident with the sites deposited with Fe [215]. When there is increased LiP in AD, the investigators evaluated the various LiPr products like malondialdehyde (MDA), isoprostanes, 4-HNE, acrolein, etc. as the identifying biomarkers at the beginning of AD [216]. The majority of the studies reported the presence of mainly MDA, isoprostanes, and 4-HNE which suggested that the deposited lipid peroxides could also be involved in the neuropathology of AD. A few of these LiPr products could also be used as a marker for the identification and prognosis of AD [217].

Several studies have also shown that the accumulation of ROS generation and decreased cortical GSH are also associated with AD pathology. Earlier investigations have shown that in AD pathology, more ROS produced whereas lowering the ROS accumulation might restore the condition in AD in the model rats [217]. In addition, these studies revealed that all the phospholipids and total fatty acids were reduced up to a significant level in the hippocampus of AD victims. Moreover, it was also observed that the AD-associated GSH levels are reduced in rats and human brain models. Zhang and their coworkers found that GSH levels have a close association with amyloidosis in the brain and the pathology of AD. So, concluded that the lipid OS (key method of ferroptosis), is intimately associated with the pathological progress of AD [218].

Several investigators have shown that Fe homeostasis and lowered endogenous antioxidant systems (along with GPX) are associated with the pathology of AD [219]. The progression of AD and cognitive decline is directly involved with the level of iron in the brain. The magnetic resonance imaging (MRI) from the affected patient showed that the affected region had a high iron quantity [220]. In comparison to a normal individual, an AD patient with mild cognitive dysfunction showed a higher amount of Fe along with an elevated Aβ plaque load in the cortical region that elevated the chances of AD [221–223]. Numerous investigators showed that an imbalance of iron in brain homeostasis is associated with Aβ plaques and NFTs [219]. Investigators also found that Fe binds directly to His6, His13, His14, and amino acid residues in β to increase the neurotoxicity of Aβ [224,225]. Studies have shown that iron regulates both the phosphorylation of τ protein and the aggregation of hyperphosphorylated τ protein [226].

Few investigators have shown that during hippocampal neurodegeneration (HND), neural death is triggered by ferroptosis in the hippocampus, which is done via ablating the forebrain neuron GPX4, which is directly correlated with cognitive impairment [227]. Some of the studies attempted in vivo experiments in mice and revealed that GPX4-deficient mice could exhibit obvious cognitive dysfunction and HND [228]. Moreover, the investigator further showed that if such mice are administered with Vit-E or lipoxstatin-1 (ferroptosis inhibitor) then there is a possibility of significant improvisation in the degree of neurodegeneration [228]. Various pieces of literature have shown that the typical preclinical signs of AD and cognitive impairment are marked by abnormal iron homeostasis, LiPr, glutathione metabolism disorder, and inflammation (trademarks of ferroptosis). Porsteinsson and their colleagues classified the clinical signs and symptoms of ADS into six stages, where stages 1 & and 2 a preclinical stage which is presymptomatic, stage 3 (prodromal): AD with MCI, stage 4 (mild AD dementia): AD with mild dementia, stage 5 (moderate AD dementia): AD with moderate dementia, and the stage 6 (severe AD dementia): AD with severe dementia. Besides this, there are certain associated symptoms/pathology with AD i.e. proof of AD pathology (Aβ and τ deposits/neural injury) throughout all the six stages. The changes in the behavioral and psychological features are observed from the second stage onwards. Cognitive impairment is observed from stage three while functional impairment is observed from stage four onwards [229].

Moreover, investigators concluded that targeted ferroptosis therapy might result in further excitotoxicity and energy deficiency. Few investigators showed that alpha-lipoic acid (LA) can help in the prevention of τ-induced iron overload, LiPr, and inflammation, which are all associated with ferroptosis [189,230,231]. A group of investigators, while working on AD, found that iron contributes a role in aggravating the polymerization of toxic Aβ and hyperphosphorylated τ. In addition to this, investigators also found that iron has a direct role in neuronal OD [232]. Iron is highly significant in ferroptosis and the pathological process of AD. Ferroptosis could help in providing new directions into the molecular pathophysiology of AD [233]. Several investigators have shown in-vivo experiments related to ferroptosis and AD in model animals like mice. A team led by Hambright exhibited that in GPX4 BIKO mice there was the deletion of GPX4, especially in neurons of the forebrain which was caused by tamoxifen. Further, the study revealed that the mice showed significant lackings in spatial learning and memory function, and HND. Finally, the team revealed that the outcomes of the experiment were involved with ferroptosis markers, for instance elevated LiPr, Extracellular signal-regulated kinase (ERK) activation, and neuroinflammation. Moreover, the investigating team supplemented the GPx4BIKO mice with a Vit-E, deficient food. Further, the team observed that there was an accelerated rate of HND and behavioral dysfunction. In addition to this, the team administered the mice with a ferroptosis inhibitor (Liproxstatin-1) and noticed improved neurodegeneration. In addition to this, in an in vitro model, iron was found to increase nerve cell death, when there was a reduction in the level of GSH levels were reduced. This happened due to the decrease in the activity of glutamic acid cysteine ligase [234]. Another study led by Hirata showed that GIF-0726-r (oxindole) stopped the cell death induced by OS (oxytosis), which was induced by glutamic acid and ferroptosis induced by erastin [235].

From both the above experiments, it was found that the excess of extracellular glutamic acid was associated with an extracellular higher iron level, which was responsible for the overactivation of glutamate receptors [236]. Due to this, there was an increased uptake of iron by the neurons and astrocytes, which resulted in the increased generation of membrane peroxides. Death of neurons, induced by glutamic acid could be mitigated by iron chelators or free radical scavengers [237]. Further, it was found that, in the excitotoxicity of glutamic acid, ferroptosis is induced by ROS [238,239]. Further, it was reported that GSH content in HT22 cell lines could be maintained by sterubin compound. During the treatment of cell lines with erastin and RSL3, the cells were protected from ferroptosis [240]. Investigators exhibited that 7-*O*-esters of taxifolin 1 and 2 have neuroprotective activity against ferroptosis induced by RSL3 in HT22 cells [241].

**Figure 8.**
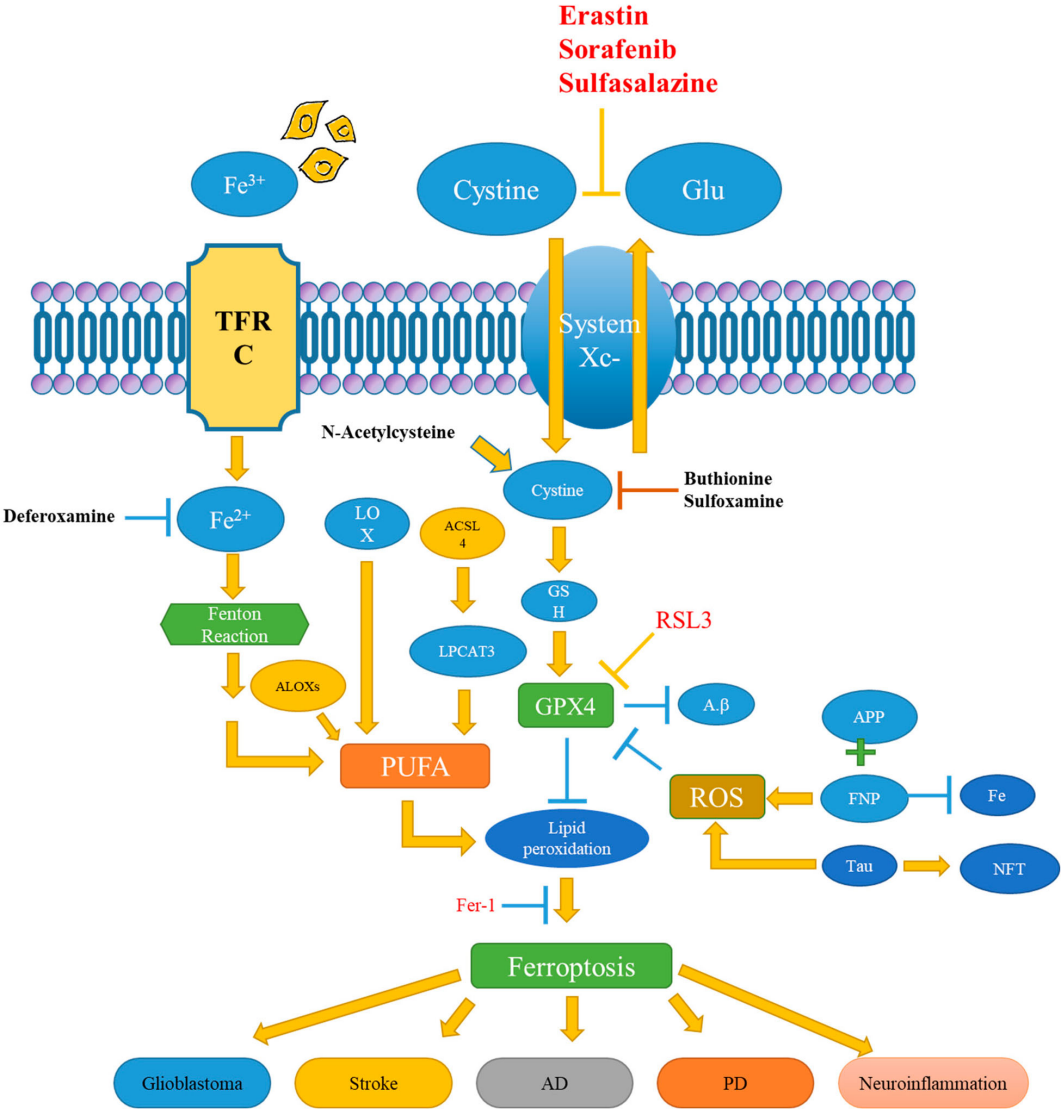
Signalling pathways of ferroptosis and associated NDDs.

An investigation showed that chalcones 14a–c successfully inhibited β-amyloid aggregation. Moreover, it was also found that the particular chalcones could provide protection to neural cells, toxicity induced by the aggregation of Aβ, and from erastin and RSL3-induced ferroptosis in human neuroblastoma SH-SY5Y cells [242]. The work depicted that toxicity induced by aggregation of Aβ plaques and of ferroptosis is inhibited because of the presence of the -OH group in the chalcones 14a–c. Moreover, it was found that the compound (chalcones 14a–c) could react with lipid peroxyl radicals by transferring H-atom, hence inhibiting LiPr [243]. A study led by Ates in mice showed that there was a reduction in the LiPr, when fatty acid synthase (FASN) was inhibited by CMS121. It was noticed that by using CMS121, there was a lesser level of 15LOX2 in the hippocampus in comparison to those of untreated WT mice. It was noticed that in the untreated ADs mice, endocannabinoids, fatty acids, and PUFAs were significantly elevated in comparison to CMS121-treated AD mice. This suggested that there might be several other enzymes that could be associated with the method of ferroptosis in AD [244]. Figure 8 shows a signaling pathway of ferroptosis and NDDs. The mechanism is briefly described below. The death of tumor cells is induced by ferroptosis by promoting the Fenton reaction which accelerates ROS generation. ACSL4 acts as an inhibitor for increase in the glioma cells by activating ferroptosis. The chemical ferroptosis inhibitors [Fer-1, Trolox an analog of (Vit-E), and deferoxamine] were found to lower the ICH-induced cytotoxicity in In vitro conditions. The examination of the brain cells in the AD victims showed biochemical and morphological properties (degradation of GSH, GPX4 inactivation, and elevated ROS) similar to ferroptosis. All these features were observed due to the imbalance of Fe homeostasis LiPr, and mitochondrial impairment. Among PD patients, an elevated progression in ferroptosis was due to Ferrostatin-1 derivatives and PKC inhibitors. The reduction in cerebrovascular damage after stroke could be achieved by using Fe chelators, antioxidants, and free radical scavengers.

Muthukumaran and their team formulated a water soluble nanomicellaer CoQ10 (Ubisol-Q10), and applied them against controlling the AD. The developed nano formulation significantly inhibited the Aβ plaque production and improvised long-term memory. So, it was concluded that ferroptosis could be a valuable process in the NDDs and AD where the ferroptosis inhibitor could play a potential and promising role in the treatment and prevention of AD [215].

The Rabies virus has a tendency to enter the CNS by crossing the BBB [245], so this concept was utilized by a team led by Nie to develop a DFO-loaded PEG-PLGA NPs with RVG29 (a rabies virus glycoprotein made up of 29 amino acid peptide) functionalization to deliver DFO to chelate large amount of Fe in the brain of PD mice [246]. A team led by Qiao developed a metal–organic framework-based nano platform which have a physical bullet-shaped structure and surface RVG29 modification which shows an excellent potential to penetrate the BBB [247].

#### 8.2.2. Parkinson’s disease (PD)

PD is a long-term chronic neurological disorder which affects the cortico-basal ganglia-thalamic circuitry [248]. It generally affects individuals over 65 years of age [249]. Every year it affects nearly 3% of elderly people and is placed second to AD [250,251]. During PD, firstly there is a deficiency of β-oxidation which causes a decrease in the long chain of acylcarnitine [56]. As a result, there is a gradual increase in the presynaptic protein α-synuclein in intracellular fibers. In the midbrain substantia nigra there is degeneration of dopamine neurons resulting in quiescent tremor, bradykinesia, and muscle stiffness [252,253].

Recent studies have shown that there is deterioration of dopaminergic nerve cells in the substantia nigra compact area (SNpc) enriched with iron, which is the major pathological feature of PD. This is the most important participant in tyrosine hydroxylase-dependent dopamine synthesis and other dopamine metabolism methods [254]. The prominent features of PD and ferroptosis are GSH depletion, LiPr, and increased levels of ROS [255]. Iron chelators like deferiprone (DFP) are known to minimize OS and enhance the activity of dopamine for improving the motor nerve clinical signs and minimizing deterioration of motor function, which results in the neuroprotective effect in the initial phases of PD [256]. Some of the work has shown the effect of iron chelators in mice models and concluded that ferroptosis is inhibited by iron chelators and these chelators protect the dopamine neurons from cell death. Moreover, the GSH level in the 1-methyl-4-phenyl-1,2,3,6-tetrahydropyridine (MPTP) mouse model was decreased [257]. As a consequence of the GSH depletion, there was an enhanced 1-methyl-4-phenyl-pyridinium ion (MPP^+^) toxicity of substantia nigra dopaminergic nerve cells [258]. All these previous studies suggested that ferroptosis is associated with the degeneration of dopamine neurons in PD. It was concluded that the inhibition of dopamine neuron ferroptosis could prove to be a successful strategy for the treatment of PD. It also observed the ferroptosis in 6-hydroxydopamine (6-OHDA)-induced PD models in SH-SY57 cells and Zebrafish. It was observed that in these models, there is a possibility of preventing 6-OHDA-induced ferroptosis after activating the p62-Keap1-Nrf2 pathway [259]. A team led by Tian showed that when 6-OHDA was used as an inducing agent for ferroptosis then the expression of FTH1 in PD rats was down-regulated significantly to control ferritinophagy, microtubule-associated protein light chain 3 and NCOA4. It was also reported that by using ferritinophagy inhibitors it is possible to inhibit the degradation of ferritin and ferroptosis induced by 6-OHDA [244,260]. During the progression of PD, ferric ammonium citrate (FAC) was used to simulate the Fe overload, and observed that lower doses of FAC were sufficient to induce ferroptosis. Moreover, when the FAC quantity was increased, then the cells mainly followed apoptosis. The above events can be rescued by the ferroptosis inhibitors by relying on regulating the p53 signaling pathway. Moreover, these above functions were not present with the apoptosis inhibitor [261].

Fuentes and their group prepared a dopamine-loaded albumin/poly lactic acid-co-glycolic acid (PLGA) nanosystems and studied them in a 6-OHDA PD mice model. It was found that the developed nanosystems efficiently crossed the BBB, and replenished dopamine at the nigrostriatal pathway which resulted in noteworthy motor symptom improvement in comparison to the lesioned and L-DOPA groups [262].

Tryphena and their group provided detailed information on the theranostic capability of the integration of miRNAs with nanotechnology. Moreover, the investigators emphasized the combined effect of both on the promises and challenges for the treatment of PDs [263].

#### 8.2.3. Glioblastoma (GBS) and brain tumors

Brain tumors could be broadly divided into 2 classes namely glioma and glioma tumors. On the basis of histopathology and degree of proliferation, glioma could be further subdivided into 4 subtypes and grades (I to IV) [264]. Grade IV glioma is commonly known as Glioblastoma [265]. GBS is a very common, invasive, aggressive, and undifferentiated type of malignant brain tumor, whose annual occurrence is 3.2 for every 100,000 persons [266–268]. The median survival of GBS patients is mainly 4–15 months from the date of diagnosis. Moreover, its prognosis is poor in addition to the higher recurrence and mortality rate (MR). The vessel in tumors has improper morphology and activity which leads to a microenvironment with lowered O_2_ tension and raised interstitial fluid pressure [269]. In addition, mitotic activity, MVC, and tumor growth factor receptors also behave abnormally in GBS. Currently, surgical removal is the most preferred treatment method for GBS patients. Since the glioma cells have a tendency of strong invasiveness a patient is required to go for adjuvant chemotherapy after surgery, which affects the clinical recovery of GBS [270]. Moreover, the efficacy of the drugs is also minimized by both types of barriers (blood–brain and blood-tumor). Though several advancements have been made for immunotherapy-based tumor treatment the treatment of brain tumors is still challenging. Most of the glioma cells overexpress the epidermal growth factor receptor (EGFR) which leads to abnormal behaviors of the underlying molecular signaling pathway [271]. Currently, EGFR and the mutant EGFRvIII are two dominant focal points in GBS therapy [272].

A number of recent studies reported NMs-assisted GBS therapy for instance ferroptosis, gene therapy (GT) [266,273], radiotherapy [274], photothermal therapy (PTT) [275], magnetothermal therapy [276,277], and immunotherapy [278]. Ferroptosis is an iron-mediated apoptosis distinguished from necrosis, autophagy [279], apoptosis, and pyroptosis [280]. Excess amount of iron reacts with H_2_O_2_ and generates free radicals and singlet O_2_ in cells. Due to the high production of free radicals, there is cytotoxic LiPr. Eventually, a combination of techniques targeting ferroptosis and apoptosis could be an effective approach to GBS treatment [57]. A team led by Yulin developed IONPs (porous, carboxyl linked) and clubbed them with GT [small interfering RNA (siRNA), GPX4, and targeting glutathione peroxidase 4] along with cisplatin and utilized the synergistic effect on the treatment of GBS-suffering patient via ferroptosis and apoptosis after surgery. This study concluded that there was a remarkable therapeutic effect with very little systemic toxicity in in-vitro and in-vivo conditions [281].

Several studies reported the use of PTT and in one such attempt, NMs with high conversion efficiency were administered and got acclimated near the tumor [282,283]. Further, that area was exposed to external irradiation which led to the generation of heat and eventually killed the tumor. A team led by Yulin developed gallic acid/ Fe^2+^ NPs with remarkable PT conservation capacity, where NIR 808 nm improved the Fe^2+^ release efficiency of NPs many folds. Investigators concluded that ferroptosis was induced in the tumor cells which also released a significant amount of heat to kill cancerous cells [281,284]. MNPs have been used for magnetothermal therapy, where NMPs are introduced to the tumor sites and upon exposure to an external magnetic field generate sufficient heat (42–45°C) to kill tumor cells. Several experiments have proven that at 42°C there is irreversible damage to tumor cells which leads to apoptosis [285,286].

More recently, Zhao and their team utilized graphdiyne (GDY) nanoplatforms for the PTT and ferroptois-based combined therapy for the treatment of GBS. GDY is one of the widely used nanomaterials due to its biocompatible nature and photothermal conversion efficiency. The investigators used FIN56 (ferroptosis inducer) for developing GDY-FIN56-RAP (GFR) polymer [287] self-assembled nanoplatforms (NPF) against GBM. The basic reason behind using GDY was the capability of the GDY to adequately load FIN56 and FIN56 discharged out from the GFR, in a pH-dependent manner. Moreover, the GFR NPF were reported to have a few advantages like penetration of BBB and acidic environment-induced in situ FIN56 discharge. In addition to this, the investigator also observed that the GBM cell ferroptosis was induced by GFR NPF by inhibiting GPX4 expression. Investigators also observed that 808 nm light rays reinforced GFR-mediated ferroptosis by raising the temperature and promoting FIN56 discharge from GFR. The investigators performed an investigation in an orthotopic xenograft mouse model of GBM, where they found that: GFR NPF was inclined to locate in tumor tissue, GBM growth was inhibited, and lifespan was increased by inducing GPX4-mediated ferroptosis. During the utilization of 808 nm rays, these GFR-mediated effects were further improved. Finally, the investigators concluded that the GFR could prove to be a suitable nanomedicine for the treatment of cancer and GFR clubbed with PTT could prove to be a promising approach against GBM [288].

Several investigators have shown the importance of various organic, polymer (nanocapsules and nanospheres), inorganic/metal (transferrin drug-loaded systems), and lipid-based (sulfatide-containing nanoliposomes) nanocarriers for the delivery of drugs against GBS, ependymoma, neuroblastoma [289], medulloblastoma, and primary CNS lymphomas. In various investigations these nanocarriers were loaded with apoptosis- and/or ferroptosis-stimulating agents which exhibited promising anti-cancer activity [290].

Manicum and their colleagues highlighted the nano-immunotherapeutic approaches for targeted RNA delivery. Further, the investigator focused on the role of monocytes/macrophages as nanocarriers for the treatment of GBS multiforme [291].

Wang and their team developed a biomimetic glioma C6 cell membrane (C6M) derived nano-vesicles (DOX-FN/C6M-NVs) loaded with doxorubicin (DOX) and iron NPs (ultra-small). This nanocarrier (DOX-FN/C6M-NVs) was further applied for the enhanced combined apoptosis and ferroptosis for the treatment of glioma. The developed nano vehicles showed enhanced therapeutic efficacy by helping in the targeted drug delivery at the tumor site and reduced cardiotoxicity and side effects of doxorubicin. The developed nanocarriers showed more potent anti-tumor activity in comparison to the free DOX-promoting/DOX-mediated apoptosis and enhanced ferroptosis via the mediation of iron NPs. Finally, the investigators concluded that the developed nanovehicle as an effective inducer of ferroptosis and apoptosis exhibited effective suppression of tumor in order to treat glioma [292].

Neuroblastoma (neural crest-derived malignancy) and meningioma are also types of brain tumors where the former accounts for more than 15% of pediatric cancer deaths [181] while the latter mainly affects old age people i.e. those over 65 years of age, and is prevalent more in women than men, and less frequently in children [293]. A team led by Hassannia claimed to identify withaferin A (WA) as a natural ferroptosis inducer in neuroblastoma. Here the investigators have used nano-sized WA which permitted systemic utilization and suppressed tumor growth because of increased accumulation at the site of tumor [181].

#### 8.2.4. Miscellaneous disorders

Mansur and their developed hybrid nano catalysts based on the conjugation of GOx (a natural enzyme) and MIONs (inorganic *nanozyme*) which was stabilized by a biocompatible polymer shell of carboxymethyl cellulose. This engineered hybrid nanocatalyst was used against cancer treatment. It was found that the magnetic IONPs (e.g. Fe_2_O_3_ and Fe_3_O_4_, MIONs) had a pH-dependent based enzymatic activity. These nanozymes could decompose hydrogen peroxide into water and oxygen by acting as catalysts under mild pH conditions. Under a mildly acidic environment, these nanozymes may use hydrogen peroxide as a substrate for the production of highly toxic ROS through the production of (°OH), exhibiting peroxidase-like activity. Here the investigators have developed supramolecular vesicle-like NMs which were evaluated for in vitro killing of tumor cells of the brain (U-87 MG) where the nanovesicle showed anticancer properties due to the ferroptosis-induced cell death. Finally, the investigators concluded that the developed hybrid NMs acted as a cascade of integrated nanocatalysts, where firstly GOx acted as a starting catalyst and generated hydrogen peroxide from the glucose in the medium. Secondly, the hydrogen peroxide (generated earlier) was catalyzed by the downstream enzymes through Fenton-like reactions generating ROS, which led to cell death [294]. A team led by Shen utilized a lactoferrin receptor-mediated transcytosis approach for the delivery of cisplatin-loaded magnetite/Gd_2_O_3_ hybrid NPs designed for ferroptosis therapy of orthotopic brain tumors [295].

## 9. Clinical trials

To date, several clinical trials are under investigation where a combined effect of ferroptosis and nanomaterials has been employed for the treatment of neurological disorders like PD, AD, GBS, and other CNS-related disorders. In most of these clinical trials, it was found that the drug carriers were a nanomaterial for better delivery of the drug. Moreover, in some of the trials, it was found that the nanocarriers were mainly made of biological material that was biocompatible, while in a few cases, the nanocarriers were porous in nature for instance (mesoporous silica NPs), which ensures a sustained and controlled release of the drug. In addition to this, some of the clinical trials have used conventional drugs like doxorubicin, dopamine, etc. in their nanosized form for enhanced uptake by the brain cells. An additional advantage of these nanosized drug/drug carriers is that they could easily cross the blood–brain barriers and may exhibit their therapeutic effect on the affected cells thus preventing the occurrence of NDDs, by inhibiting the ferroptosis. In one of the clinical trials involvement of ferroptosis’ in dopaminergic cell death was confirmed in the MPTP mouse for PDs. Mostly, iron and iron oxide-based NPs have been used for inducing ferroptosis-based therapy for NDDs. In addition to this, several non-iron-based nano-ferroptotic inducers have also been used (silica NPs, carbon dots) for inducing ferroptosis, for instance, the best one is carboxyl-modified polystyrene nanoparticles (CPS). It was found that the CPS could gain access to cells via micropinocytosis and could effectively protect the cells from ferroptosis by lowering the intracellular ROS and triggering the lysosome stress in a size-dependent fashion [296].

## 10. Conclusions

Ferroptosis-mediated cell death is still in its infancy stage and very little information is available in the domain. But, in recent years numerous attempts have been made in this field, due to the participation of ferroptosis in several neurodegenerative disorders like Parkinson’s and Alzheimer’s disease, and glioblastoma. Most neurodegenerative disorders have an association with iron accumulation in the brain. Ferroptosis mainly involves iron metabolism, LiPr, and the cystine/glutamate system. Experimental studies carried out in vivo have established several mechanisms involved in ferroptosis. Several attempts have shown the effect of ferroptosis inducers and inhibitors on several neurodegenerative disorders. There is a requirement for more potent and specific nano-ferroptotic drugs, the rational combination of ferroptotic treatment with another anti-CNS-related disorder approach for synergistic efficacy, and the fabrication of novel-anti ferroptosis NPs for the treatment of NDDs. This field might still result in promising and disruptive therapeutic alternatives for patients suffering from neural disorders.

**Abbreviations**
ACSL4: Acyl-CoA synthetase long-chain family member 4
AD: Alzheimer’s Disease
DAMP: Damage-Associated Molecular Pattern
ERK: Extracellular Regulated Kinases
FTH1: Ferritin heavy chain 1
GAPDH: Glyceraldehyde 3-phosphate dehydrogenase
GBS: Glioblastoma
GPX4: Glutathione peroxidase 4
GSH: Glutathione
GSSG: Oxidized glutathione
HND: Hippocampal NeuroDegeneration
IOGNPs: Iron oxide glyconanoparticles
IRPs: Iron Regulatory Proteins
LiPr: Lipid Peroxidation
LOX: Lipoxygenases
MRI: Magnetic resonance imaging
NADPH: nicotinamide adenine dinucleotide phosphate
NFE2L2: erythroid 2-related factor 2
NMs: Nanomaterials
NDDs: Neurodegenerative disorders
OS: Oxidative Stress
PD: Parkinson’s disease
PEs: Phosphatidylethanolamine
PUFAs: Polyunsaturated fatty acids
PUF-CoA: Polyunsaturated fatty acids- Acetyl coenzyme A
ROS: Reactive oxygen species
TFR 1: transferrin receptor 1
VDACs: Dysfunction of varistor anion channels

## Data Availability

All the data are present within the manuscript.
